# Preliminary study on the efficacy of xerostomia treatment with sialocentesis targeting thyroid disease patients given radioiodine therapy

**DOI:** 10.1186/s40902-019-0223-3

**Published:** 2019-09-05

**Authors:** Euy-Hyun Kim, Dong-Keon Lee, Chang-Woo Kim, In-Seok Song, Sang-Ho Jun

**Affiliations:** Department of Oral and Maxillofacial Surgery, Korea University Anam Hospital, 73, Inchon-ro, Seongbuk-gu, Seoul, Republic of Korea

**Keywords:** Sialocentesis, Radioiodine, Submandibular gland, Sialendoscopy, Xerostomia

## Abstract

**Background:**

Radioiodine therapy has been widely used for thyroid disease patients, but hyposalivation and xerostomia may occur in 10~30% of patients. Sialocentesis is a procedure that removes inflammatory substances in the salivary duct and expands the duct for the secretion and delivery of saliva. In this study, thyroid disease patients treated with radioactive iodine were selected among the patients with xerostomia who visited the hospital, and the effect of sialocentesis was compared and analyzed. And then, comparison between the radioiodine therapy-experienced group and the non-radioiodine therapy-experienced group was conducted.

**Results:**

In this study, we studied xerostomia patients who underwent radioiodine therapy due to thyroid diseases and who underwent sialocentesis at the Korea University Anam Hospital. Sialocentesis is conducted by one surgeon. The study also compares the clinical symptoms before and after the surgery. After the procedure, the discomfort due to xerostomia was reduced, and the symptom was improved effectively.

**Conclusions:**

The results of this study showed that sialocentesis has a clinical effect in the treatment of xerostomia, which is a side effect of radioiodine therapy. In addition, the possibility of further clinical application of sialocentesis in the future is found.

## Background

Radioiodine (^131^I) therapy has been widely used for thyroid disease patients. Radioiodine is used for the treatment of thyroid cancers and is concentrated in the salivary gland. The amount of radioiodine in the salivary gland is 20~100 times more than that in serum. Ten to 30% of patients have complications of decreased salivary gland functions, due to damage of the salivary parenchyma from radiation. A number of symptoms can occur, such as dry mouth, hyposalivation, pain of the oral cavity of the parotid/submandibular region, and altered taste. Both dose of radioiodine and time of concentration can affect the extent of symptoms [1–4].

Sialocentesis is a procedure that removes inflammatory substances in the salivary duct, such as mucous plugs and other debris. The procedure is gradual. After dilation of the salivary duct, the duct is washed by 20~30 ml of saline, and the diluted dexamethasone-saline solution is used. This procedure can be repeated additionally, which depends on the severity of symptoms of the patients. Post-operative swelling of the major salivary gland can occur; the symptoms diminish as time goes by. After the procedure, regular follow-ups are proceeded to compare the subjective complaints of the patients.

By using a microscope and sialendoscopy in sialocentesis, the accuracy and successibility of operation can be enhanced significantly. They enable the operator to detect an orifice of salivary gland ducts, which leads to high success rates of the procedure. According to Kim, obvious causes such as stenosis and lesion in ducts are best indications of using sialendoscopy [5–8].

In this study, thyroid disease patients treated with radioiodine were selected among the patients with xerostomia who visited the hospital. Among this group, the clinical effect of sialocentesis was compared and analyzed. 

## Methods

**Fig. 1:**
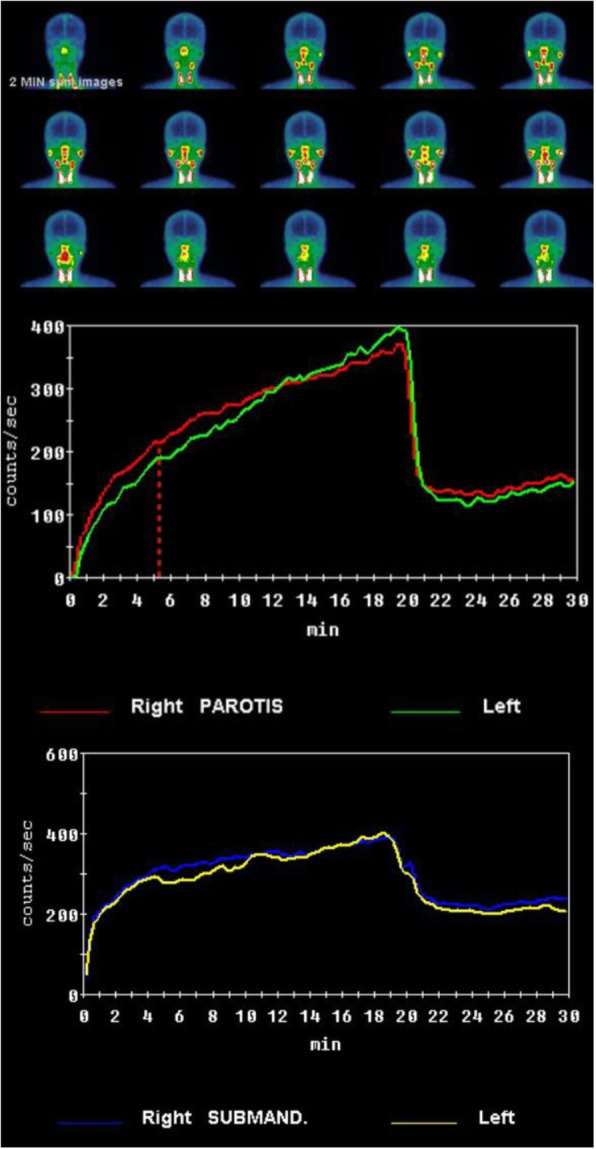
Salivary gland scintigraphy

**Fig. 2:**
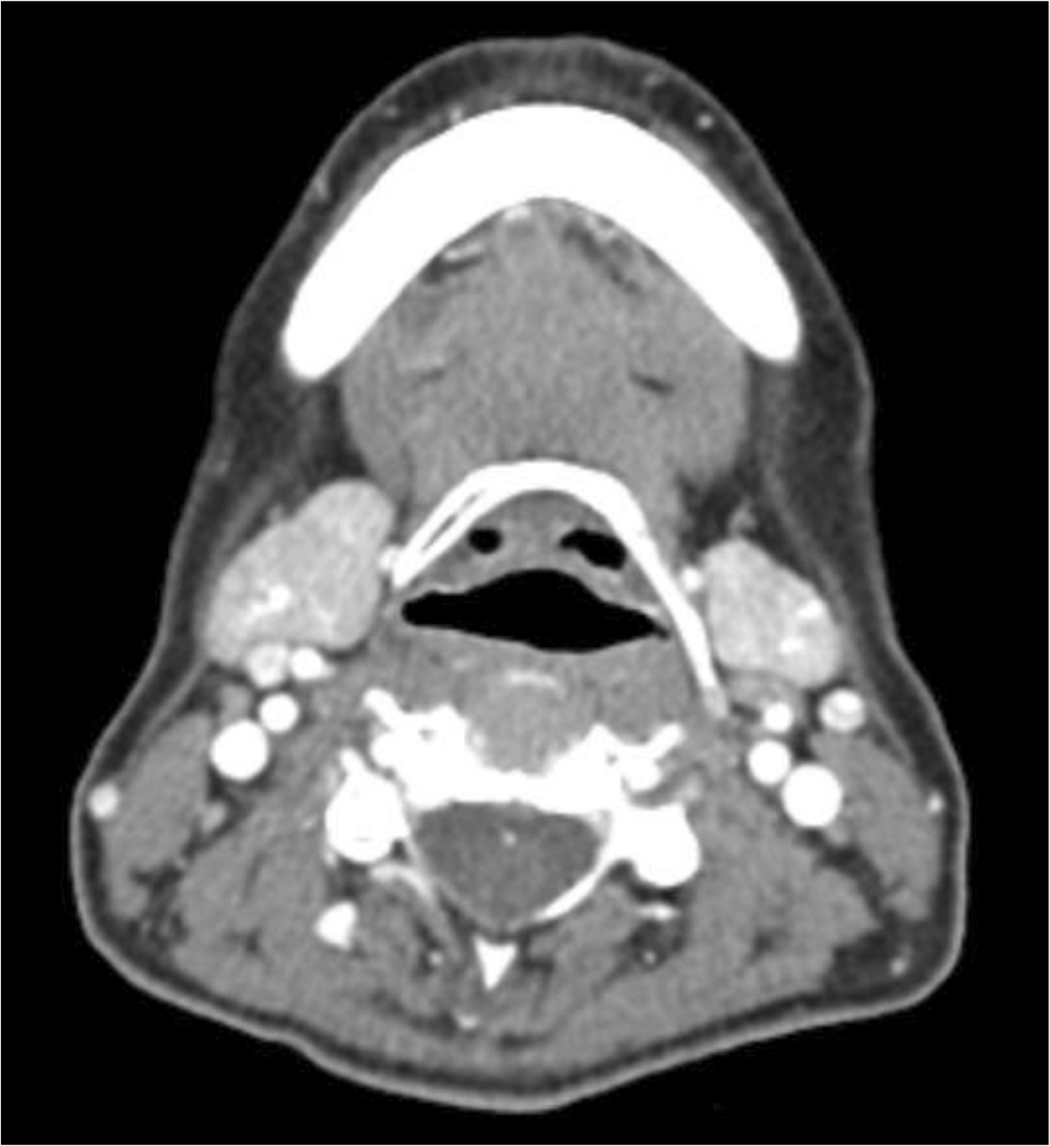
Neck CT

The aim of the study is analyzing the efficacy of xerostomia treatment with sialocentesis which is the procedure of enlarging the salivary duct, especially Wharton’s duct. Clinical procedures consisted of four visits. In the first visit, a general and oral examination is proceeded. Salivary gland scintigraphy (Fig. 1) and neck CT (contrast-enhanced, Fig. 2) are taken. Sialography shows the salivary flow rate of the major salivary glands, especially the parotid gland and submandibular gland. It indicates how severe the xerostomia of a patient is, and it helps in determining the treatment procedure. Neck CT shows the deformity or atrophy of the salivary gland. Contrast-enhanced image is needed for detailed information. Also the first visual analogue scale (VAS) record was taken. Pre-operative scintigraphy shows the flow rate of the saliva from both the parotid gland and submandibular gland. Tc^99m^ (Technetium ppm pertechnetate) was used for taking scintigraphy. Salivary flow rate graph can compare between submandibular and parotid glands, also between the left and right glands. In the second visit, a record of gland scan and CT in each visit was identified to compare between pre-operative score and post-operative score [9]. Salagen (Pilocarpine, Eisaikorea) prescription was done in 0.5 TAB, TID for 2 months to enhance natural salivary flow rate. We use this medication after identifying record of gland scan and CT image. In the third visit, sialocentesis was done in the operation room. Local anesthesia is an option for pain-sensitive patients, and the third VAS was taken. At last, in the fourth visit, a 1-week recall check was done and post-operative VAS was taken.

Four patients who underwent radioiodine therapy associated with thyroid diseases are selected. Clinical assessments and treatment procedures are conducted in the Department of Oral and Maxillofacial Surgery, Anam Hospital, Korea University. Clinical procedures are performed by one surgeon.

In this study, we compared the VAS score associated with each clinical visit, maximum probe size, and dexamethasone dose used in irrigating salivary ducts. Also we experimented our patient group who have taken radioiodine therapy (four patients), comparing with the non-radioiodine therapy group (95 patients) who underwent sialocentesis in the Department of Oral and Maxillofacial Surgery, Anam Hospital, Korea University.

In the operation room, after extraoral and intraoral drapping, we try to find the orifice of the submandibular and parotid glands with the microscope. Approach of both gland duct with #0000 probe is done, and gradual enlargement with as large as the possible probe. The expansion size of the duct depends on stenosis or inflammation of salivary ducts. After the enlargement phase, 24-Gauge catheter insertion on ducts of both glands was done. Through this catheter, irrigation of both the salivary duct with saline and saline/dexamethasone 9:1 solution (either dexamethasone 0.5 mg or 1 mg) was done. After the irrigation, post-operation caution was explained to patients, such as gentle massage of both salivary glands extraorally and hot pack application on the face [3, 4, 7].

## Results

**Fig. 3:**
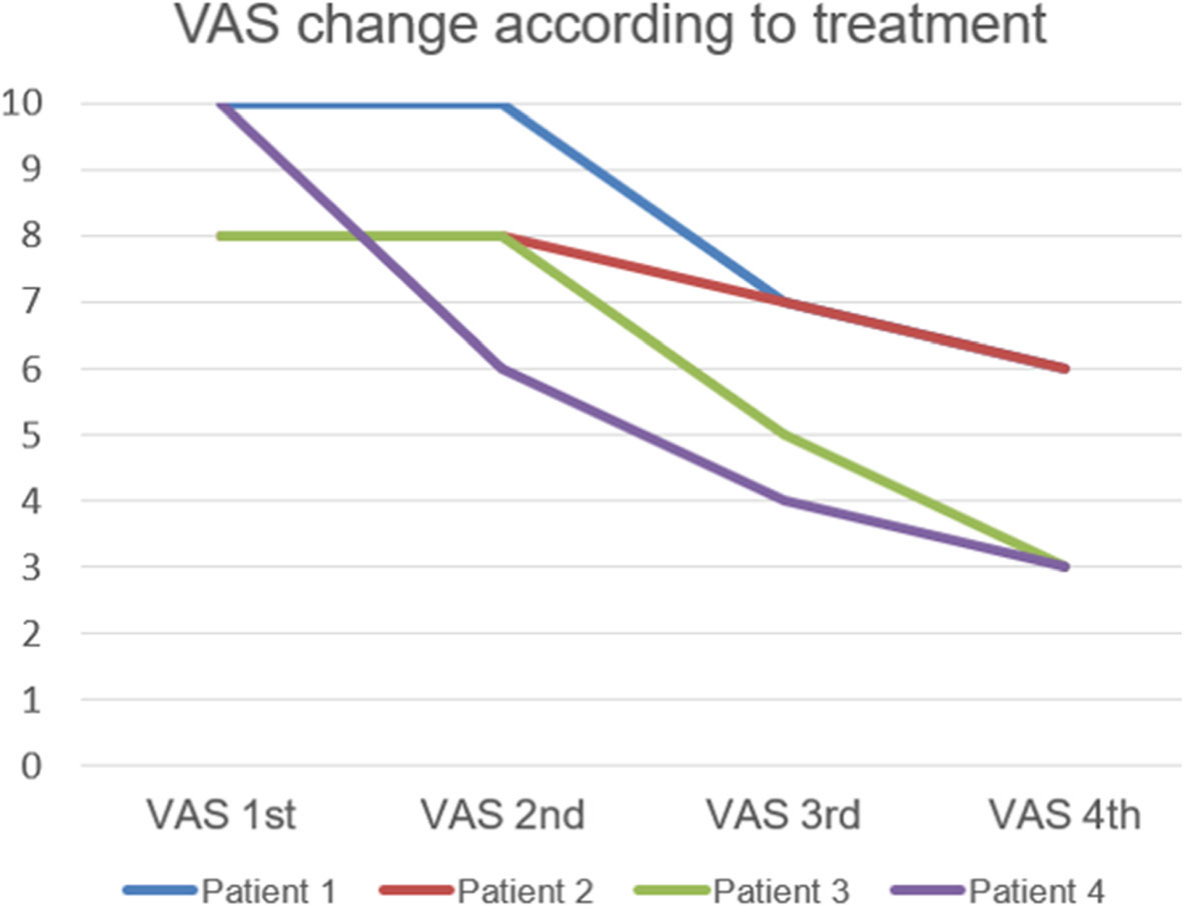
VAS change according to the treatment type

Before and after the procedure, the VAS score was taken and compared (Fig. 3). The biggest decline in VAS score was found between the second and third VAS. The maximum VAS score was 10, and the minimum VAS score was 3. All results of the patient group indicated that VAS score had declined as the treatment time passed by.

**Fig. 4:**
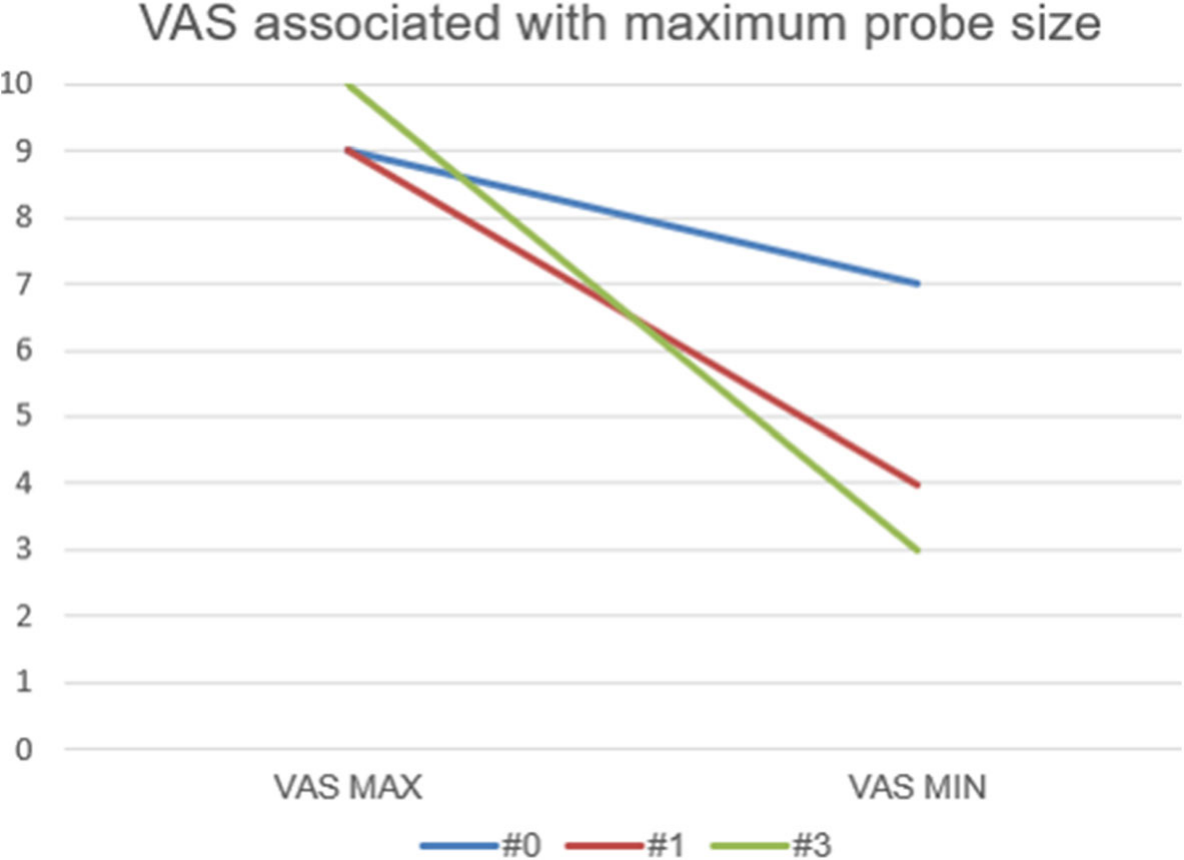
VAS associated with the maximum probe size

The biggest decline in VAS score was associated with the probe size used in sialocentesis (Fig. 4). This figure implies the maximum probe size that was used during the procedure. In that point, “#0” means that the #0 probe was used lastly in the procedure. The bigger the probe used in the procedure, which exceeds #3 size, the more decline was found after the procedure. In the case of probe #0, a decline of VAS score from 10 to 8 was found. Meanwhile, in the case of probe #3, a decline of VAS score from 9 to 3 was found. These results indicated that the more enlarged salivary duct can decrease the discomforts of patients.

**Fig. 5:**
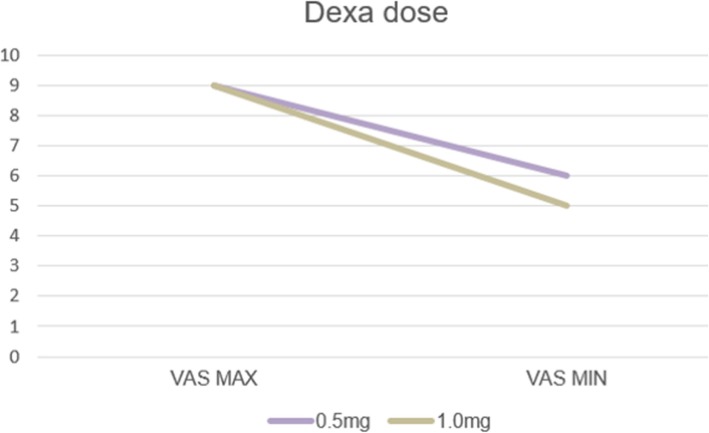
VAS associated with dexamethasone dose

From the point of view of dexamethasone dose, even the result is unremarkable, and a difference existed (Fig. 5). There were two choices of dexamethasone dose, 0.5 mg and 1.0 mg. In the case of the 0.5-mg dose of dexamethasone, a decline of VAS score from 9 to 6 was found. Similarly, in the case of the 1.0-mg dose of dexamethasone, a decline of VAS score from 9 to 5 was found.

**Fig. 6:**
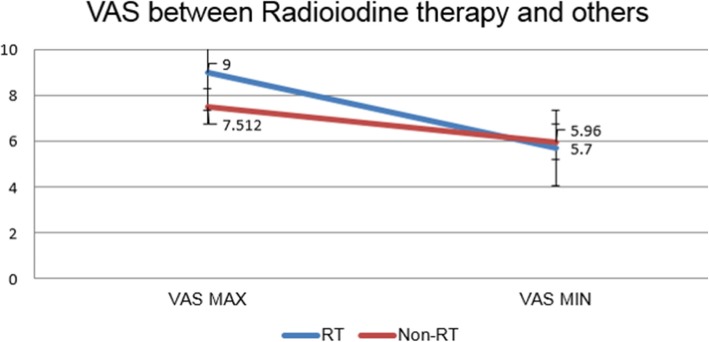
VAS between radioiodine therapy and others

Finally, a comparison between the radioiodine therapy-experienced group and the non-radioiodine therapy-experienced group was conducted (Fig. 6). VAS scores included in the comparison were limited to the first VAS. In the case of the radioiodine therapy-experienced group, a decline of VAS score from 9.00 to 5.70 was found. Similarly, in the case of the non-radioiodine therapy-experienced group, a decline of VAS score from 7.51 to 5.96 was found.

## Discussion

Routinely, scintigraphy was taken in every patient without exception. Scintigraphy can help the operator to analyze and compare both salivary glands in terms of function. This information can enhance the success rate of sialocentesis. In this study, we had not taken scintigraphy after the procedure. That means other studies about comparing pre- and post-operative records are required. The post-operative records can indicate the clinical success of sialocentesis [2, 5].

In this study, selection and investigation were done between the radioiodine therapy-experienced group (4/99) and the non-radioiodine therapy-experienced group (95/99). Selection criteria of both groups were experience and non-existence of radioiodine therapy history. More detailed criteria can be applied in selecting patient groups, such as onset period and other medical histories [1, 3, 6].

The onset of xerostomia is a critical parameter of diagnosis. The period of discomfort-related experience shows how severe are the symptoms and illnesses. Moreover, treatment methods and guidelines could be changed upon the discomfort period of each patient. In this study, the onset of patient was omitted which means that further evaluation of each patient’s onset period is required [2].

General medical history is related to the diagnosis and treatment of xerostomia. In our clinic, 21/99 patients (21.2%) have hypertension and 14/99 patients (14.1%) have diabetes. Thyroid-related disease is in 15/99 patients (15.2%), and cancer is in 16/99 patients (16.2%). In terms of these results, more detailed studies about the correlation of these diseases with xerostomia are required. Radioactive therapy aimed at cancer patients is also known as the cause of dryness of the mouth and mucositis, which is a major complication [4].

Pre-operative scintigraphy was taken routinely for evaluating the saliva-secreting function of the major salivary gland of the patients. Due to the short study period, we did not take any post-operative sialographies. In the near future, we are planning to conduct post-operative scintigraphy and further studies comparing pre- and post-operative scintigraphy [9].

Sjogren’s syndrome was known as causing symptoms such as xerostomia and dry eye syndrome. Generally, an antibody screening test including factor SSA was routinely performed to diagnose Sjogren’s syndrome. If the syndrome exists, treatment goal can be changed from xerostomia. Steroid or antirheumatoidal drugs can be considered to relieve the discomfort of patients [3].

Due to few patients—four patients—were included in the study, this study was limited in terms of quantity. Larger range and quantity of patients should be included in further studies. Moreover, a week of the follow-up period was too short for accurate investigation. Longer follow-ups were required for monitoring patient’s event and conditions. In that period, post-operative scintigraphy can be taken [2, 8].

The effect of sialocentesis depends on the condition of the patients, but normally, it lasts for 6 months. There is a tendency that the salivary flow rate decreases just after the procedure. In this study, less than 1 more week follow-ups were taken. More detailed studies are required for evaluating the long-term efficacy of sialocentesis [10].

## Conclusions

The results of this study showed that sialocentesis has a clinical effect in the treatment of xerostomia, which is a side effect of radioiodine therapy. In point of view of VAS, a significant decrease is found during the study. Further clinical applications and studies are required for more detailed results.

## Data Availability

Please contact the author for data requests.

## References

[1] Mandel SJ, Mandel L (2003). Radioactive iodine and the salivary glands. Thyroid.

[2] Rigler R, Scanlon P (1955) Radiation parotitis from radioactive iodine therapy. In: Proceedings of the staff meetings. Mayo Clinic 14371749

[3] Solans R, Bosch J-A, Galofré P, Porta F, Roselló J, Selva-O’Callagan A (2001). Salivary and lacrimal gland dysfunction (sicca syndrome) after radioiodine therapy. J Nuclear Med.

[4] De Luca R, Vicidomini A, Trodella M, Tartaro G, Colella G (2014). Sialoendoscopy: a viable treatment for I131 induced sialoadenitis. Br J Oral Maxillofac Surg.

[5] Bomeli SR, Schaitkin B, Carrau RL, Walvekar RR (2009). Interventional sialendoscopy for treatment of radioiodine-induced sialadenitis. Laryngoscope.

[6] Wu C-B, Xi H, Zhou Q, Zhang L-M (2015). Sialendoscopy-assisted treatment for radioiodine-induced sialadenitis. J Oral Maxillofac Surg.

[7] Kim JW, Han GS, Lee SH, Lee DY, Kim YM (2007). Sialoendoscopic treatment for radioiodine induced sialadenitis. Laryngoscope.

[8] Lee D-K, Kim E-H, Kim C-W, Kang M-H, Song I-S, Jun S-H (2019). Sialolithotomy of the submandibular duct using sialendoscopy. Maxillofac Plast Reconstr Surg.

[9] Grove AS, Di Chiro G (1968). Salivary gland scanning with technetium 99m pertechnetate. Am J Roentgenol.

[10] Karagozoglu KH, Vissink A, Forouzanfar T, Brand HS, Maarse F, Jager DHJ (2018). Sialendoscopy enhances salivary gland function in Sjögren’s syndrome: a 6-month follow-up, randomised and controlled, single blind study. Ann Rheumatic Dis.

